# Associations Between Dust Storms and Intensive Care Unit Admissions in the United States, 2000–2015

**DOI:** 10.1029/2020GH000260

**Published:** 2020-08-01

**Authors:** C. S. Rublee, C. J. Sorensen, J. Lemery, T. J. Wade, E. A. Sams, E. D. Hilborn, J. L. Crooks

**Affiliations:** ^1^ Department of Emergency Medicine University of Colorado School of Medicine Aurora CO USA; ^2^ United States Environmental Protection Agency Chapel Hill NC USA; ^3^ Division of Biostatistics and Bioinformatics National Jewish Health Denver CO USA; ^4^ Department of Epidemiology Colorado School of Public Health Aurora CO USA; ^5^ Now at National Jewish Health Main Campus Denver CO USA

**Keywords:** climate change, dust storm, health care utilization, health, respiratory, critical care

## Abstract

Anthropogenic climate change is influencing the incidence of dust storms and associated human exposure to coarse particulate matter (PM_2.5–10_) in the United States. Studies have found adverse health consequences related to dust exposure. These consequences include respiratory disease exacerbations and premature mortality, resulting in increased health care utilization. However, the impact of dust storms on critical care demand has not been studied in the United States. We seek to quantify the relationship between dust storms and surges in critical care demand by investigating the association between dust storms and intensive care unit (ICU) admissions at nearby hospitals from 2000 to 2015. ICU data were acquired from Premier, Inc. and encompass 15–20% of all ICU admissions in the United States. Dust storm, meteorology, and air pollutant data were downloaded from the U.S. National Weather Service, the U.S. National Climatic Data Center, and the U.S. Environmental Protection Agency websites, respectively. Associations between ICU admission and dust storms, controlling for temperature, dew point temperature, ambient PM_2.5_ and ozone, as well as seasonally varying confounders, were estimated using a distributed lag conditional Poisson model with overdispersion. We found a 4.8% (95% CI: 0.4, 9.4; *p* = 0.033) increase in total ICU admissions on the day of the dust storm (Lag 0) and a 9.2% (95% CI: 1.8, 17.0; *p* = 0.013) and 7.5% (95% CI: 0.3, 15.2; *p* = 0.040) increase in respiratory admissions at Lags 0 and 5. North American dust storms are associated with increases in same day and lagged demand for critical care services at nearby hospitals.

## Key Points

- Climate change is influencing dust storms and adversely impacting health, especially for vulnerable populations
- We found North American dust storms are associated with increases in same day and lagged demand for critical care services at nearby hospitals
- Public health and health care systems need to prepare for the health impacts of climate change now

## 1 Introduction

Anthropogenic climate change is expected to impact future exposure to airborne particulate matter (PM) associated with ambient dust and dust storms, with potentially costly human health consequences. According to the Environmental Protection Agency's (EPA) Climate Change Impacts and Risk Analysis (CIRA) framework, future impacts from airborne dust ranks fourth in terms of projected economic impacts (Achakulwisut et al., 2019). Recent droughts and heat waves have reached record intensity in some regions of the United States, and on a global scale there is evidence of resulting increased frequency of dust storms (USGCRP, 2018; Wehner et al., 2017). Desertification is expected to worsen by the end of the century without substantial reductions in greenhouse gas emissions (IPCC, 2018). Drought, high temperatures, and resulting decreases in surface soil moisture create ideal conditions for dust storms (Wehner et al., 2017).

Ambient dust contains a heterogenous mixture of suspended particles including PM_2.5_ (PM with aerodynamic radius less than 2.5 μm), PM_2.5–10_ (radius 2.5–10 μm), as well as organic matter, human pathogens, pollen, and anthropogenic pollutants, which are also particles and therefore parts of PM, each with unique human health impacts (Gonzalez‐Martin et al., 2014). During dust storm events, PM levels may exceed national and international acceptable levels and may remain suspended in local environments for hours to days in the United States and even longer on other continents (Goudie, 2014). Additionally, ambient dust may travel thousands of kilometers (km) and affect downwind populations (Goudie, 2014). Observations indicate that in arid geographic regions, dust may constitute up to 50% of monthly PM_2.5_ and 75% of PM_10_ levels (Hand et al., 2017). Abundant epidemiological studies have established that both short and long‐term exposure to PM is associated with negative health consequences, including premature mortality, decreased lung function, exacerbation of respiratory disease, and a broad range of adverse cardiovascular impacts and adverse birth effects (Guaita et al., 2011; US EPA, 2009, 2018).

Globally, dust storms have been found to be associated with increased health care utilization with varying degrees of lagged health impacts. Dust storm exposure has been shown to increase emergency department visits (Cadelis et al., 2014; Tam et al., 2012; Thalib & Al‐Taiar, 2012; Wehner et al., 2017), hospitalizations (Bell et al., 2008; Chiu et al., 2008; Grineski et al., 2011; Kim et al., 2015; Lee et al., 2008; Merrifield et al., 2013; Prospero et al., 2008; Samoli et al., 2011), and outpatient visits (Q. Zhang et al., 2013) across a broad range of age groups, medical diagnoses, and geographic regions. However, the specific impact of dust storms on the demand for critical care services is currently unknown. Intensive care units (ICUs) care for the most seriously ill patients and require copious resources, uniquely trained staff, and specialized equipment. Unexpected surges in demand for these limited resources can cause a cascade of stresses on hospital operations. To quantify the relationship between dust storms and surges in critical care demand, we investigated the association between dust storms at a hospital's ZIP code and ICU admissions at that hospital. By analyzing hospital data and storm data nationally over time, we sought to account for the heterogeneity of dust storm composition and community level factors that limit single‐storm studies. Such information can help fill knowledge gaps regarding the health impacts of North American dust storms and assist regional public health officials, hospital managers, and emergency planners prepare for current and future climate scenarios.

## 2 Materials and Methods

### 2.1 ICU Admissions

ICU admissions data were acquired from Premier, Inc., a medical data aggregation company, whose hospitalization database encompasses 15–20% of all admissions in the United States (Premier Allied Sciences, 2019). Cardiac, medical, surgical, and pediatric ICU data were included. The data were acquired in 2019 and provide records from 1 January 2000 to 31 December 2015. All 48 contiguous U.S. states were included. Admissions were classified based on the *International Classification of Diseases, 9th Revisions* (ICD‐9). All‐cause ICU admissions include all ICD‐9 codes, cardiovascular admissions include 390–448, and respiratory admissions include 480–486, 490–497, or 507. Admissions that had both cardiovascular and respiratory codes listed were included in both categories. Daily counts of ICU admissions for all, respiratory, and cardiovascular causes were summarized by the ZIP code of the hospital where the ICU admission occurred. Counts for all‐cause ICU admissions were further broken down by age, gender, and race. Counts for all categories were broken down by year and U.S. Census Division.

### 2.2 Dust Storms

There is no clear consensus worldwide or in the United States regarding how dust storms should be classified. Previous studies have used monitored PM_10_ concentrations, ratios between PM_10_, PM_2.5_ and species' concentrations, visibility, ground‐based aerosol optical depth measurements, satellite imagery, and meteorological models. Technical challenges limit the use of solely particulate concentrations, especially at high concentrations, for accurate classification of dust storms.

Following Crooks et al. (2016), the present study used dust storms as reported in the U.S. National Weather Service (NWS) storm database, specifically events with the EVENT_TYPE listed as “Dust Storm.” The database is the most complete storm record for the United States, aggregating storm reports from the general public, insurance industry, law enforcement, NWS damage surveys, emergency management officials, and others (https://www.ncdc.noaa.gov/stormevents/faq.jsp). Six Saharan dust incursions reported in Puerto Rico and the U.S. Virgin Islands were dropped in order to focus on dust associated with storm systems rather than with long‐range dust transport. Two dust storms reported east of the Mississippi (in Indiana and Delaware) were also dropped. Our final dust storm data set included 967 storm events in the years 1996–2017 and 819 storm events in the years 2000–2015. The former interval allowed characterization of long‐term trends, while the latter interval was used in the ICU analysis.

Individual dust events listed by weather forecast zone (WFZ) were associated with specific hospital ZIP codes if the WFZ and ZIP code intersected or if the minimum distance between them was 20 km or less. The impact of varying this buffer distance is explored in a sensitivity analysis.

### 2.3 Air Pollution and Meteorological Data

Ambient monitor‐based meteorology and air pollutant data were downloaded from the U.S. National Climatic Data Center (https://www.ncdc.noaa.gov/) and the U.S. EPA (https://www.epa.gov/outdoor-air-quality-data) websites, respectively. These data included 24‐hr averages (in the local time zone) of temperature, dew point temperature, PM_10_ and PM_2.5_ concentrations, and ozone mixing ratios for 2000–2015. Monitor‐based PM and ozone data that are not reported to the EPA Air Quality System were not included in the analysis. As in previous work (Abdo et al., 2019), we calculated daily values of the air pollution and meteorological variables at each ZIP code in the United States by taking the median of all monitors reporting data on the given day that were located within the ZIP code or within a 20 km buffer of the ZIP code centroid.

### 2.4 Merging Data

Air pollution, meteorology, dust storm, and ICU data were merged together by ZIP code and date. However, only those ZIP codes and dates that could contribute to estimating the dust storm association under the time‐stratified conditional Poisson model used in the ICU analysis below were retained. The stratum for a given dust storm in a given ZIP code included the date of the dust storm as well as a set of control days defined as dates within the same year and 3‐month season (January–March, April–June, etc.) as the dust storm and falling at 28‐day intervals from the dust date. Thus, each stratum contained a dust storm day and two to three control days. This approach enabled automatic control for time‐invariant factors (such as location), slowly varying factors (such as population changes), and day‐of‐week effects. Days falling up to 5 days after the dust storm day or control days were also included in the stratum to allow analysis of lagged health impacts. Thus, the median number of days in a stratum was 18. While uncommon, it was possible for a ZIP code to experience multiple dust storms in a single season; strata were then merged. The median number of dust storms in a stratum was 1, but a few strata included more (up to 6). A total of 32,909 strata were created containing 569,174 individual stratum days of which 34,790 were impacted by a dust storm.

Merging strata with ICU data was performed by Premier, Inc. based on date and hospital ZIP code. The final merged data set contained 1,831 unique strata. This number is lower than before because many dust‐impacted ZIP codes in the United States do not include a hospital whose data are included in the Premier database. However, those strata that remained contained 30,538 stratum days of which 1,994 were impacted by a dust storm.

To protect the confidentiality of data providers, Premier restricted location and date information in the data set released to the authors to a randomized stratum identifier (i.e., strata were defined by ZIP code and date but these labels were stripped out in the final data set), the day number within the stratum (1, 2, …), the year, and the U.S. census region.

### 2.5 Dust Storms, Meteorology, and Air Pollution

To understand the meteorological and air pollution correlates of the dust storm reports in the NWS database, and to confirm that the reports reflect a true increase in ground‐level PM levels, we estimated associations between the meteorology and air pollution variables and same‐day dichotomous dust storms. Specifically, we used mixed effects regression models and random intercept by stratum and nonlinear control for time‐of‐year using a natural spline on date with 8 degrees of freedom per year. Models were fitted using the lme function in the lme4 package (Bates et al., 2015). All analyses in the paper were performed using R (R Core Team, 2017) version 3.6.0.

### 2.6 Dust Storms and ICU Admissions

To characterize associations between ICU admissions and dichotomous dust storms, controlling for temperature, dew point temperature, ambient PM_2.5_, and ozone, as well as slowly varying confounders, we used distributed lag time‐stratified conditional Poisson models (Armstrong et al., 2014). Associations were estimated using the gnm function of the gnm package (Turner & Firth, 2018) with the quasipoisson family selected to account for potential overdispersion of the ICU counts. Individual lagged associations for Lags 0–5 were calculated and then averaged over 0–2, 3–5, and 0–5 lag day windows using the glht function of the multcomp package (Hothorn et al., 2008). Temperature at Lags 0 and 1 and dew point temperature at Lag 0 were modeled using natural splines with 3 degrees of freedom. Slowly varying confounders were modeled using a natural spline on date with 8 degrees of freedom per year. Precipitation was not included in the ICU models due to the lack of association with dust storms, while PM_10_ was not included because of its high collinearity with dust storms.

### 2.7 Sensitivity Analyses

Further analyses were performed to evaluate the sensitivity of our ICU model results to changes in our confounder model, data processing workflow, and lag period. First, nine alternative confounder models were fitted, each of which included a different subset of the variables (i.e., temperature, dew point temperature, time, PM_2.5_, and ozone) from the main model (see Table S1 in the supporting information). Several of the confounder models (e.g., the dust storm only model) are clearly not appropriate to the goal of the present work but are included for the sake of comparison. Second, to check whether the distributed lag model was overparameterized, the lag period was shortened to 3 and 4 days instead of 5 days in the main model. Third, to assess the impact of varying the buffer distances used to assign exposures to ZIP codes, the buffer distance used to assign meteorology and air pollution observations was varied from 20 to 10 and 50 km, while the buffer distance used to assign dust storms was varied from 20 to 10 km.

## 3 Results

### 3.1 ICU Admissions

The counts of ICU admissions in our data set are broken out by cause, age, race, and sex, cross‐referenced by year and region (Table 1). There were just over twice as many cardiovascular admissions as respiratory (some admissions were counted in both groups), and ICU admissions with at least one cardiovascular code accounted for 78% of total ICU admissions. The 60–79 year age group had the highest ICU admission counts across age groups, with 41% of the total, while Caucasians had the highest across race groups (79%). Men were admitted to the ICU at higher rates than women (55% vs. 45%). ICU admissions were more common in the latter years of the study (93% of admissions occurred in the period 2011–2015). It is uncertain whether this reflects an increase in dustiness, an aging population, or a change in reporting. Admissions were also heavily weighted toward the mountain region (91%).

**Table 1:** Admitted ICU Patient Characteristics Based on Age, Sex, and Race and Characterized by Time (2000–2005, 2006–2010, and 2011–2015) and Region (Central, Mountain, and Pacific)

		Time			Region		
Group	Total	2000–2005	2006–2010	2011–2015	Central	Mountain	Pacific
Cardiovascular	26,332	898	898	24,536	281	23,952	2,099
Respiratory	11,714	299	416	10,999	130	10,677	907
All ICU Admissions	33,679	1,143	1,224	31,312	361	30,689	2,629
**Age in years (%)** ^a^							
0–19	1,500 (4)	20	26	1,454	27	1,384	89
20–39	4,085 (12)	102	149	3,834	37	3,771	277
40–59	9,473 (28)	287	381	8,805	81	8,693	699
60–79	13,841 (41)	519	488	12,834	154	12,573	1,114
80+	4,780 (14)	215	180	4,385	62	4,268	450
**Sex (%)** ^a^							
Male	18,544 (55)	622	667	17,255	203	16,909	1,432
Female	15,135 (45)	521	557	14,057	158	13,780	1,197
**Race (%)** ^a^							
African American	1,963 (6)	37	57	1,869	27	1,820	116
Caucasian	26,638 (79)	909	977	24,752	270	24,209	2,159
Other race	5,078 (15)	197	190	4,691	64	4,660	354

^a^ Counts include all ICU admissions.

### 3.2 Dust Storms

**Figure 1:**
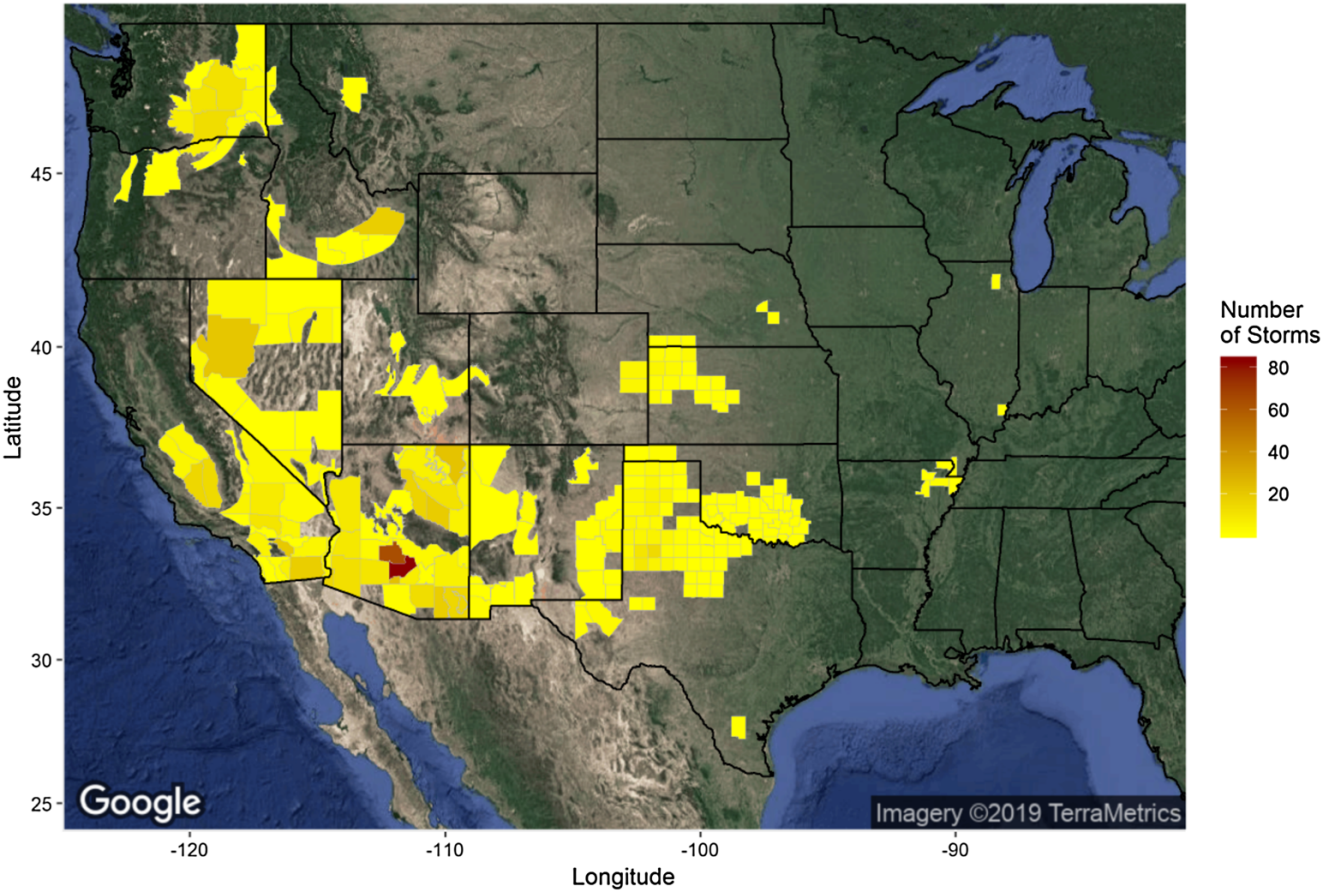
National Weather Service forecast zones (WFZ) colored by the number of reported dust storms (1996–2017) observed. Zones without dust storms are not colored. Satellite imagery was downloaded from Google Maps (Google, Inc) on 14 March 2019 and mapped using the ggmap package in R.

Dust storms were reported throughout the western United States (see Figure 1). The majority of WFZs had 40 or fewer dust storms over the study period with the maximum reaching almost 80 observed storms in parts of Arizona. Similar patterns were observed when mapping the observed storms by ZIP code (see Figure S1). However, it should be noted that there are areas of the country that are known to experience dust storm activity but where no dust storms are reported NWS storm database, for example, west Texas and the San Luis Valley in southern Colorado.

**Figure 2:**
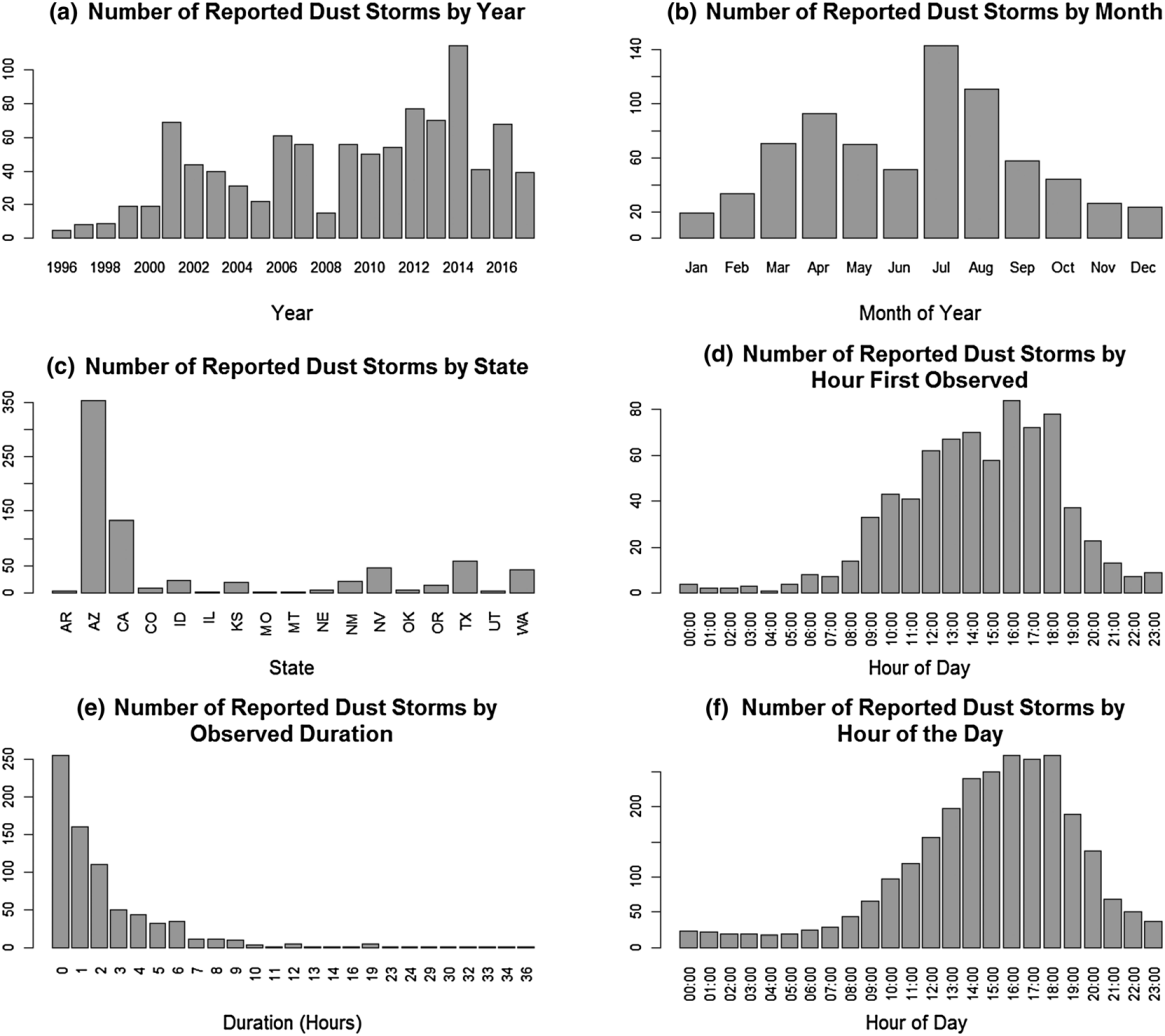
The number of reported dust storms (1995–2017) in the United States (as listed in the National Weather Service storm database) by (a) year, (b) month, (c) state, (d) hour of the day of initial storm observation, (e) observed duration, and (f) hour of the day with ongoing storm. Due to reporting errors or biases, the number of storms reported to the NWS may not represent the true number of dust storms that occurred in the United States during the study period.

Figure 2 summarizes other aspects of the dust storm data. Similar to Crooks et al. (2016), which reported dust storm events in the years 1993–2010, we found an overall increasing trend in dust storm reports from 1996 to 2017 (Figure 2a). We also find that the national intra‐annual distribution of dust storms is bimodal, with the highest numbers reported in July and August and a smaller bump seen in April (Figure 2b). As indicated by Figure 1, Arizona had the most dust storms (*N* = 353), followed by California (*N* = 133), Texas (*N* = 58), Nevada (*N* = 47), and Washington State (*N* = 43) (Figure 2c). Dust storms tended to be first observed in the early and midafternoon (Figure 2d) and last only a few hours (Figure 2e) by local time. Thus, an ongoing dust storm was most likely to be observed in the afternoon (Figure 2f).

### 3.3 Air Pollution and Meteorological Data

Summary statistics for the ZIP code‐level meteorological and air pollution data in all strata used in this study are given in Table 2, which includes the statistics calculated over all days, dust storm days only, and non‐dust storm control days (Table 2). Statistically significant (*p* < 0.05) differences between dust and non‐dust storm days are apparent for all variables except precipitation. Mixed effects model associations between weather and air pollution variables and dust storms at Lag 0 are presented in Table 3. All variables except precipitation and ozone show a statistically significant positive association with dust storms. Ozone displays a negative association (approximately −0.52 ppb), while precipitation shows no association. Average temperature and dew point temperature, however, are both 2.3–2.4°F higher on dust storm days. Furthermore, PM_10_ has a strong positive association, being 47.8 μg/m^3^ higher on dust storm days, while PM_2.5_ is somewhat higher at 3.7 μg/m^3^. This confirms that dust storm events in the NWS database do at least on average reflect a true increase in airborne dust.

**Table 2:** Summary Statistics of Meteorological and Air Pollution Variables During All Days in the ICU Analysis Including Dust Storm Days, Control Days, and Lag Days

		All days	Dust storm	
			No	Yes
**Mean temperature (°F)**				
	Mean (SD)	82.8 (14.6)	82.3 (14.7)	89.9 (11.3)
	Min, Max	25.9, 105.4	25.9, 105.4	48.5, 104.3
**Dew point temperature (°F)**				
	Mean (SD)	46.7 (14.3)	46.3 (14.4)	52.9 (11.6)
	Min, Max	−2.2, 72.2	−2.2, 72.2	10.8, 71.7
**Precipitation (tenths of inches)**				
	Mean (SD)	0.3 (2.5)	0.3 (2.6)	0.2 (1.2)
	Min, Max	0.0, 116.6	0.0, 116.6	0.0, 13.4
**PM**_**2.5**_ **(μg/m** ^**3**^ **)**				
	Mean (SD)	8.9 (6.5)	8.7 (6.3)	12.0 (8.3)
	Min, Max	0.3, 107.8	0.3, 107.8	2.6, 62.0
**PM**_**10**_ **(μg/m** ^**3**^ **)**				
	Mean (SD)	35.3 (24.4)	32.9 (19.1)	67.9 (50.6)
	Min, Max	5.0, 589.0	5.0, 230.5	13.0, 589.0
**Ozone (ppb)**				
	Mean (SD)	51.4 (12.5)	51.2 (12.7)	54.2 (9.5)
	Min, Max	6.0, 98.0	6.0, 98.0	19.0, 86.0

*Note*. Mean, standard deviation (SD), minimum, and maximum are shown.

**Table 3:** Associations Between Meteorological and Air Pollution Variables and Dust Storm Events Controlling for Time of Year

	Association point estimate	*p* value	95% CI
O_3_ concentration (ppb)	−0.52	0.0194	−0.95, −0.08
PM_2.5_ concentration (μg/m^3^)	3.7	2.0 × 10^−118^	3.4, 4.0
PM_10_ concentration (μg/m^3^)	47.8	0	46.2, 49.3
Precipitation (inches)	−0.0085	0.071	−0.0176, 0.0007
Average temperature (°F)	2.38	2.5 × 10^−83^	2.14, 2.62
Dew point temperature (°F)	2.34	1.3 × 10^−35^	1.97, 2.71

*Note*. Associations were estimated using mixed effects models with fixed nonlinear time‐of‐year effects and random intercept by season‐within‐year and ZIP code.

### 3.4 Dust Storms and ICU Admissions

**Figure 3:**
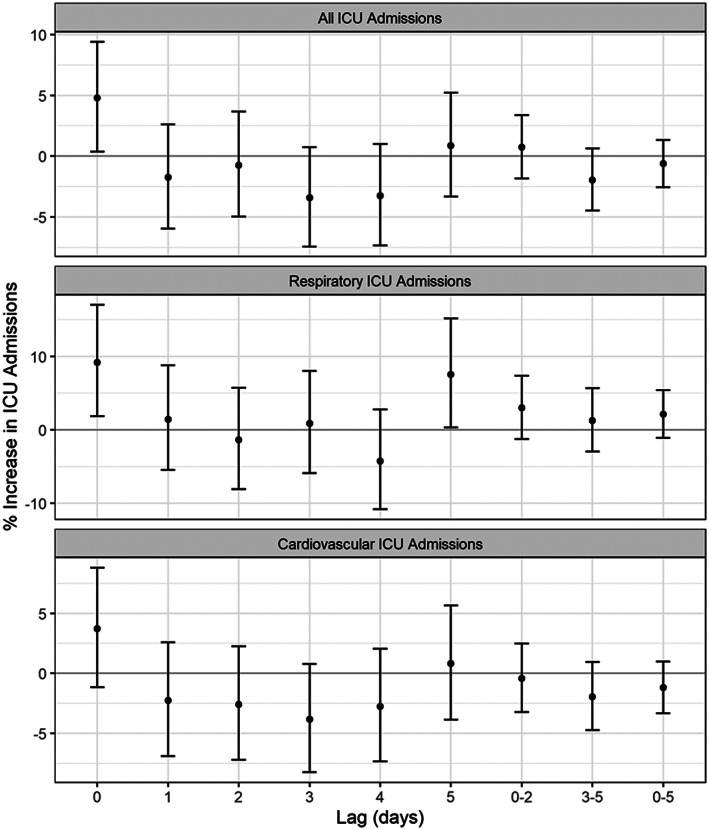
Percent increase in ICU admission risk associated with lagged dichotomous dust storm event from distributed lag models for ICU admissions (all, respiratory, and cardiovascular) for the years 2000–2015. Respiratory admissions fall under ICD‐9 codes 480–486, 490–497, or 507, while cardiovascular admissions fall under 390–448.

Results from our main model show a significant increase in ICU admissions with dust storms for all and respiratory causes (see Figure 3). Specifically, the models yielded a 4.8% (95% CI: 0.4, 9.4; *p* = 0.033) increase in total ICU admissions on the day of the dust storm (Lag 0) and a 9.2% (95% CI: 1.8, 17.0; *p* = 0.013) and 7.5% (95% CI: 0.3, 15.2; *p* = 0.040) increase in respiratory admissions at Lags 0 and 5, respectively. No associations with cardiovascular admissions were found. However, the overall pattern of association over lags was similar across the endpoints, with the highest associations (statistically significant or not) found at Lags 0 and 5, possibly reflecting the overlap in counts between the groups.

### 3.5 Sensitivity Analyses

Results from alternative confounder models are shown in Figure S2, which include averages of single lag day associations as 0–2, 3–5, and 0–5. The model corresponding to the main results above is colored magenta and is the rightmost interval at each lag. Of the nine alternative models presented, eight models reproduce the positive association at Lag 5, five reproduce the positive association at Lag 0 for respiratory admissions, and six recapitulate the positive association at Lag 0 for all admissions. The models that fail to confirm our main results were those that either did not include any meteorology or air pollution variables, or those that included temperature, dew point temperature, and PM_2.5_, but not ozone. However, given the associations in Table S1 and the known relationships between these variables and health, the most appropriate model is the one that includes all of them.

The differences in confidence interval widths between the models appear to be driven by sample size, with the models incorporating PM_2.5_ having the widest intervals. In many places, PM_2.5_ is monitored only every third or sixth day, which decreases the number of usable stratum days. Sample sizes across models are given in Table S1. However, including ozone in the model drops the sample size even further than including PM_2.5_ but does not increase the confidence interval width as much, perhaps reflecting the stronger association between PM_2.5_ and dust storms.

The impact of changing the number of lag days in the distributed lag models are explored in Figures S3 and S4, which give results for models with lagged effects out to 3 and 4 days, respectively. Dropping the distal lags has little impact on the remaining lag associations; positive associations at Lag 0 are still found for all and respiratory admissions. The similarity among distributed lag models reflects the low autocorrelation in the dust storm exposure due the paucity of dust storms lasting more than a few hours.

Figure S5 shows that increasing the buffer distance for assigning weather and air pollution observations to ZIP codes from 20 to 50 km has little impact on our results, yielding the same set of associations as found in our main model. However, decreasing the buffer distance to 10 km buffer distance eliminated several associations; only the Lag 5 association with respiratory admissions remained. This difference is due in part to the wider confidence intervals estimated when using the 10 km buffer compared to the others, which is itself driven by the lower sample size. Table S2 shows the number of ICU admissions under the three buffers. Decreasing the buffer distance to 10 km cut the number of ICU admissions by more than half, whereas increasing the buffer to 50 km increased the number of admissions by approximately 10%. Finally, Figure S6 compares the main results while varying the buffer distance used to assign dust storms to ZIP codes. Table S3 shows the impact of varying the WFZ buffer distance on these counts. Decreasing this buffer distance from 20 to 10 km increased the width of the confidence intervals (though not as drastically as with the monitor buffer distance above), leaving only the Lag 0 association with respiratory admissions still rejecting the null. This is due to a loss in sample size by about 10% under the smaller buffer.

## 4 Discussion

This is the first national‐scale study of dust storms and morbidity in the United States. We found a 4.8% (95% CI: 0.4, 9.4; *p* = 0.033) increase in total ICU admissions on the day of the dust storm (Lag 0) and a 9.2% (95% CI: 1.8, 17.0; *p* = 0.013) and 7.5% (95% CI: 0.3, 15.2; *p* = 0.040) increase in respiratory admissions at Lags 0 and 5, respectively. No statistically significant associations with cardiovascular admissions were found. Results contribute to a growing body of evidence showing that dust exposure affects health and health care utilization.

To model the potential impact on ICU admissions from a hypothetical severe dust storm, we apply the dust storm‐PM_10_ association of 47.8 μg/m^3^ from Table 3 and assume a severe dust storm with a 24‐hr PM_10_ concentration of 400 μg/m^3^ similar to other studies (Al‐Taiar & Thalib, 2014; Draxler et al., 2001). By comparison, the maximum 24‐hr PM_10_ ZIP code concentration included in the present study was 589 μg/m^3^, and four ZIP code days featured 24‐hr concentrations exceeding 400 μg/m^3^. Under a 400 μg/m^3^ dust storm scenario, the all‐cause ICU admissions at Lag 0 increase 48.0% (95% CI: 3.3, 112.1), a substantial jump in ICU admissions over a short period of time. Respiratory ICU admissions at Lags 0 and 5 increase significantly as well. Respiratory ICU admissions increase 108.4% (95% CI: 16.4, 272.0) at Lag 0 and 83% (95% CI: 2.6, 226.0) at Lag 5.

Such an acute increase in demand for critical care services would challenge and potentially overwhelm many ICUs and health care systems. Applying the respiratory ICU admissions from this study, a hypothetical hospital that usually has 20 ICU admissions per day total would increase to more than 40 admissions. The increase would likely contribute to ICU strain, or at least perceived strain by health care teams. The baseline acuity of patients, census, and time of admissions may impact quality of patient care provided, provoke early discharges, and contribute to less job satisfaction (Kerlin et al., 2014; Rewa et al., 2018). In addition, this scenario exacerbates projected shortages of ICU specialists, with the needs of our aging population already expected to lead to ICU supply and demand mismatches (Angus et al., 2000).

The risk for ICU admissions during dust storms was highest at Lag 0 for all ICU and respiratory admissions. The results are consistent with expected acute respiratory inflammatory responses related to particles and associated toxins or pollutants being carried with the particles (Zhang et al., 2016). The increased association found at Lag 5 for respiratory admissions may be attributed to several causes. There may be a delay in presentation initially with management at home or in the outpatient setting until worsening status or access to care becomes available. Several infections have also been shown to be associated with dust exposure such as meningitis and influenza, which take days to incubate in the human body before symptom presentation (Schweitzer et al., 2018; Zhang et al., 2016).

The majority of patients included in the study were from recent years (2011–2015). There were more reported dust storms during recent years consistent with trends of increased frequency of dust storms (USGCRP, 2018; Wehner et al., 2017). However, ICU numbers may also reflect changes in the composition of the Premier database. The sample obtained from Premier, Inc. may have had more patient data in later years simply due to an overall increase in partners in the southwestern United States. Finally, components of dust storms such as toxins or other pollutants may be worsening and contributing to increased health care needs (Buzea et al., 2007; Yamada et al., 2012).

Results from North American studies are consistent with numerous global epidemiological studies of dust storms and airborne dust. While estimated associations are not always consistent between studies (likely due to differences in the composition of dust, susceptibility of the exposed population, magnitude of the exposure, choice of health outcome and study design, and differences in preventative measures adopted by communities; Crooks et al., 2016; Zhang et al., 2016), many have similarly found positive associations between exposure to dust storms and morbidity related to respiratory and all‐cause morbidity (Crooks et al., 2016; Goudie, 2014; Zhang et al., 2016). For example, hospital admissions for respiratory concerns increased in Kuwait on the day of the dust storms similar to the present study (Thalib & Al‐Taiar, 2012), and a systematic review on the acute respiratory health effects, including asthma, bronchitis, pneumonia, and chronic obstruction pulmonary disease, demonstrated similar results (Zhang et al., 2016).

Studies have been carried out in many regions of the world, including southern Europe (Díaz et al., 2012; Faustini et al., 2015; Karanasiou et al., 2012; Mallone et al., 2011; Middleton et al., 2008; Neophytou et al., 2013; Perez et al., 2008; Reyes et al., 2014; Stafoggia et al., 2016; Tobías et al., 2011; Trianti et al., 2017), east Asia (Chiu et al., 2008; Higashi et al., 2014; Kanatani et al., 2010; Kurai et al., 2017; Lee et al., 2008; Lee, 2013, 2014; Nakamura et al., 2016; Nakao et al., 2018; Watanabe et al., 2015, 2016; Yoo et al., 2008), the Middle East (Al‐Taiar & Thalib, 2014; Khaniabadi et al., 2017; Neisi et al., 2017; Thalib & Al‐Taiar, 2012; Vodonos et al., 2014, 2015), the Caribbean (Cadelis et al., 2014; Gyan et al., 2005; Prospero et al., 2008), and Australia (Barnett et al., 2012; Johnston et al., 2011; Merrifield et al., 2013; Rutherford et al., 1999). However, despite high dust activity in many U.S. states, the United States has lagged behind other regions in the study of dust storms and their health effects. While there have been a few local‐scale epidemiological studies (Grineski et al., 2011; James et al., 2018) of dust storms in the United States since the year 2000, there have only been two large‐scale studies of dust storms and population health published in the United States (Crooks et al., 2016; Tong et al., 2017).

Results from this study support a growing body of literature demonstrating life‐threatening and costly health consequences associated with environmental conditions that are expected to increase under all future climate scenarios (Schweitzer et al., 2018; UNEP et al., 2016; USGCRP, 2016, 2018). By 2080–2099, the U.S. Southwest is projected to experience coarse dust increasing up to 38% with asthma emergency department visits increasing 88% (Achakulwisut et al., 2019), with accompanying health costs increasing an additional $47 billion per year on top of the $13 billion per year already being spent to treat dust‐related illness (Achakulwisut et al., 2019). ICU admissions are important to monitor as they are expensive to individuals and society as a whole, with the first day of an ICU admission costing thousands of dollars (Dasta et al., 2005) and can lead to cascading system‐wide stress.

There is an urgent need for environmental scientists and health professionals to work together to mitigate the effects of dust storms on human health. Improved technologies that accurately measure and predict storms as well as a better understanding of dose‐response relationships and the identification of vulnerable populations may allow for the creation of early warning systems and preemptive hospital preparation. Dust storm mitigation may also alleviate current and future health impacts, notably through sustainable land development strategies and land preservation, which have been described but with inconsistent implementation and action plans (Chen & Cai, 2003; Middleton, 2017; Schweitzer et al., 2018; Sternberg & Edwards, 2017). Further efforts may expand to study indirect health effects of dust storm via influence on energy systems, transportation, infrastructure, ecological systems, and health care systems.

The study has several strengths. Data obtained represents 15–20% of ICU admissions across the nation, with a high concentration of patients represented in the regions where dust storms occur. We were able to use the ICD codes for all causes in addition to respiratory and cardiovascular admissions to better characterize results. Six variables (temperature, dew point temperature, precipitation, ambient PM_2.5_, PM_10_, and ozone) were controlled for to reduce bias. Our results were robust to most changes in the confounder model and data processing steps, with the results for respiratory ICU admission at Lag 0 being particularly robust.

A few limitations remain. First, dust storms were characterized using observations reported by the National Weather Service, which performs no standardized quality review on its storm reports. However, we found empirically that dust storms were associated with an increase of nearly 50 μg/m^3^ in 24‐hr PM_10_ concentrations in those ZIP codes with nonmissing PM_10_ data. Second, it was assumed that patients would seek care at their nearest hospital location. We lacked ZIP code data for patients and could not identify their location when dust storms occurred. With travel, the study results would underestimate the true burden of critical illness by not capturing all affected individuals and misclassifying exposure. Third, data limitations only allow analysis of a subset of all ICU admissions in the nation, with a greater proportion of individuals in the latter years, potentially introducing selection bias. Fourth, because ICU admissions can have both respiratory and cardiovascular ICD‐9 codes our results for our three end points are not independent. Fifth, dust storms tend to occur in the afternoon and evening, while ICU admissions occur all day; thus, the positive association we found at Lag 0 may underestimate the true post‐storm ICU impact. Sixth, much remains to be discovered about the toxicological components of PM and other factors associated with dust storms that may modify or confound associations between ICU admissions and dust storm exposures.

## 5 Conclusion

We found positive associations between North American dust storms and increased ICU admissions in the vicinity of the storm for respiratory and all‐cause morbidity. Results contribute to a growing body of evidence supporting a myriad of adverse health effects resulting from dust exposure. These results suggest that public health prevention and health care system readiness is urgently needed now and in the future to buffer negative impacts to vulnerable patients and health care systems resulting from surge in demand for critical care services.

## Conflict of Interest

The authors declare no conflict of interest relevant to this study.

## Supporting information



Supporting Information S1

## Data Availability

Data supporting our conclusions can be obtained from the Open Science Framework website at https://osf.io/2jpmz (Crooks, 2020).
