# Supplementary material for: Associations Between Dust Storms and Intensive Care Unit Admissions in the United States, 2000–2015
Source: Geohealth. 2020 Aug 1;4(8):e2020GH000260. doi: 10.1029/2020GH000260 (PMC7411550; doi:10.1029/2020GH000260)

Supporting Information for

**Associations Between Dust Storms and Intensive Care Unit Admissions in the United States,**

**2000-2015**

C. S. Rublee, MD, MPH,^1^ C. J. Sorensen, MD,^1^ J. Lemery, MD,^1^ T. J. Wade, PhD, MPH,^2^ E. A. Sams,^2^ E. D. Hilborn, DVM, MPH, Dipl. ACVPM,^2^ and J. L. Crooks, PhD, MS^3,4^

^1^ University of Colorado School of Medicine, Department of Emergency Medicine, Aurora, CO

^2^ United States Environmental Protection Agency, Chapel Hill, NC

^3^ National Jewish Health, Division of Biostatistics and Bioinformatics, Denver, CO

^4^ Colorado School of Public Health, Department of Epidemiology, CO

**Contents of this file**

Tables S1 to S3

Figures S1 to S6

**Introduction**

The supporting information contains additional details of results from the main manuscript.

Table S1: Number of ICU admissions under different confounder models. Count totals vary due to differences in the number of complete cases when variables with missing values are included in the models.

| Confounder Model | All ICU Admissions | Respiratory ICU Admissions | Cardiovascular ICU Admissions |
| --- | --- | --- | --- |
| Dust Storm | 47350 | 15960 | 36896 |
| Dust Storm + Temp + Dew Point | 46689 | 15729 | 36336 |
| Dust Storm + Temp + Dew Point + PM_2.5_ | 39385 | 13467 | 30616 |
| Dust Storm + Temp + Dew Point + O_3_ | 36688 | 12581 | 28739 |
| Dust Storm + Temp + Dew Point + PM_2.5_ + O_3_ | 33679 | 11714 | 26332 |
| Dust Storm + Time | 47350 | 15960 | 36896 |
| Dust Storm + Temp + Dew Point + Time | 46689 | 15729 | 36336 |
| Dust Storm + Temp + Dew Point + PM_2.5_ + Time | 39385 | 13467 | 30616 |
| Dust Storm + Temp + Dew Point + O_3_ + Time | 36688 | 12581 | 28739 |
| Dust Storm + Temp + Dew Point + PM_2.5_ + O_3_ + Time | 33679 | 11714 | 26332 |

Table S2: Number of ICU admissions in strata with complete cases under different buffer distances used to assign monitored air pollution and meteorological data to ZIP codes. The main model results correspond to the 20km buffer.

| Monitor Buffer Distance | All ICU Admissions | Respiratory ICU Admissions | Cardiovascular ICU Admissions |
| --- | --- | --- | --- |
| 50km | 36373 | 12563 | 28457 |
| 20km | 33679 | 11714 | 26332 |
| 10km | 16576 | 5813 | 13004 |

Table S3: Number of strata, stratum days, and dust days before and after merging Premier ICU data, by WFZ buffer distance.

|  | WFZ Buffer | # Strata | # Stratum Days | # Dust Days |
| --- | --- | --- | --- | --- |
| Pre-Merge | 20km | 32909 | 569174 | 34790 |
|  | 10km | 27011 | 469489 | 28435 |
| Post-Merge | 20km | 1831 | 30538 | 1994 |
|  | 10km | 1541 | 25942 | 1666 |

Figure S1: ZIP Codes colored by the number of reported dust storms (1996-2017) assigned. ZIP codes without assigned dust storms are not colored. Satellite data were downloaded from Google Maps (Google, Inc) on March 18, 2019 and mapped using the ggmap package in R. ZIP codes overlapping or falling within 20km of a dust-impacted WFZ (Figure 1) were treated as dust-impacted themselves.


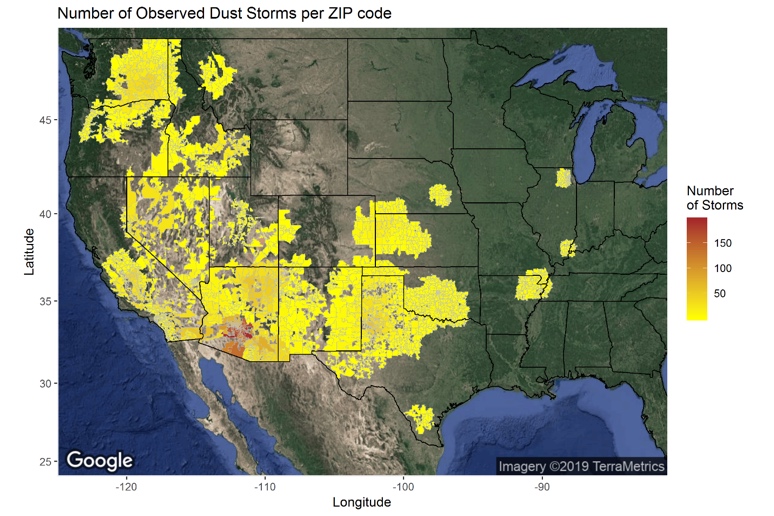


Figure S2: Alternative confounder models. Our primary model (Dust Storm + Temp + Dew Point + PM2.5 + O3 + Time) results are shown in magenta as the right-most interval at each lag.


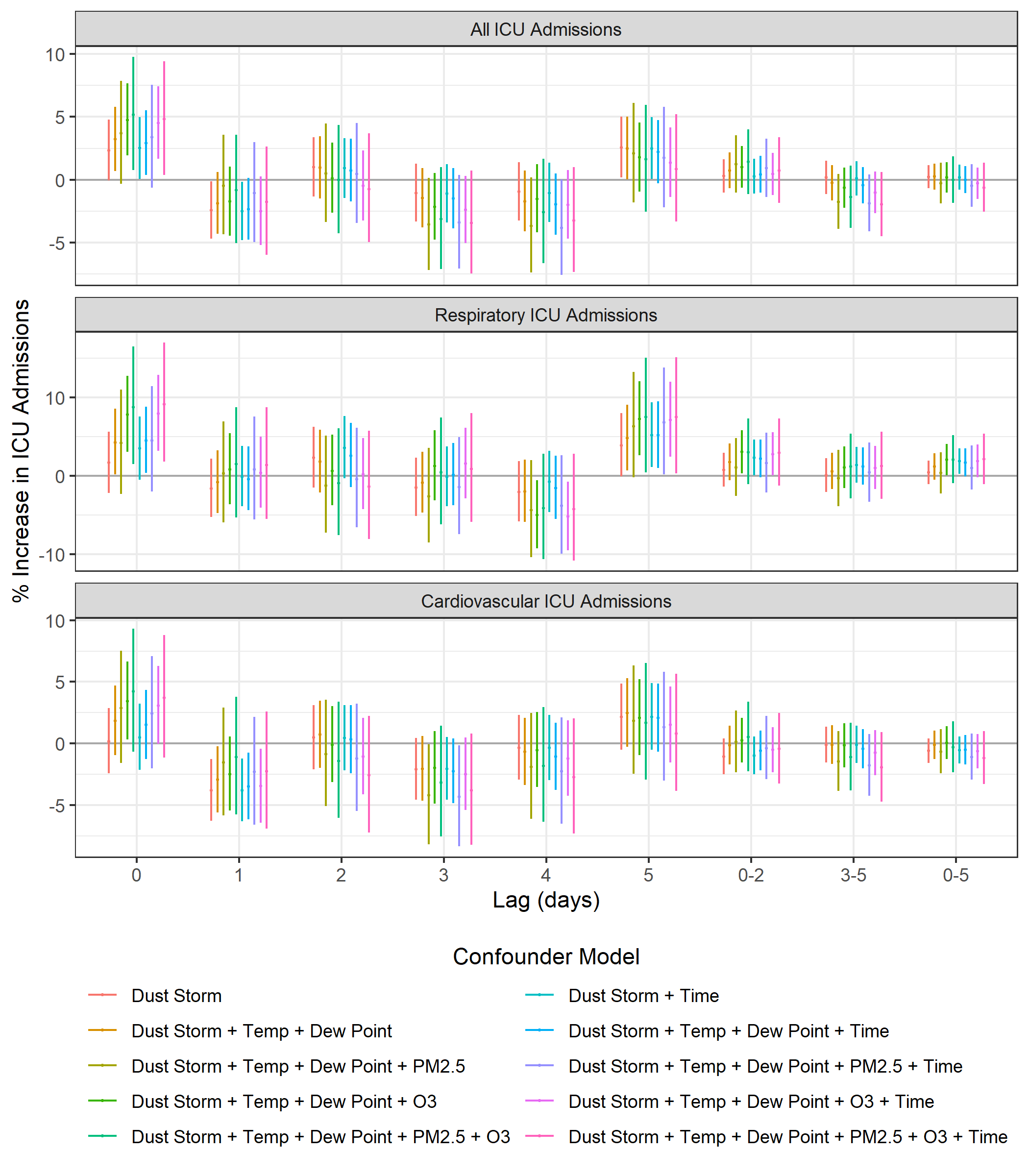


Figure S3: Lagged effects modeled at 0-3 days.


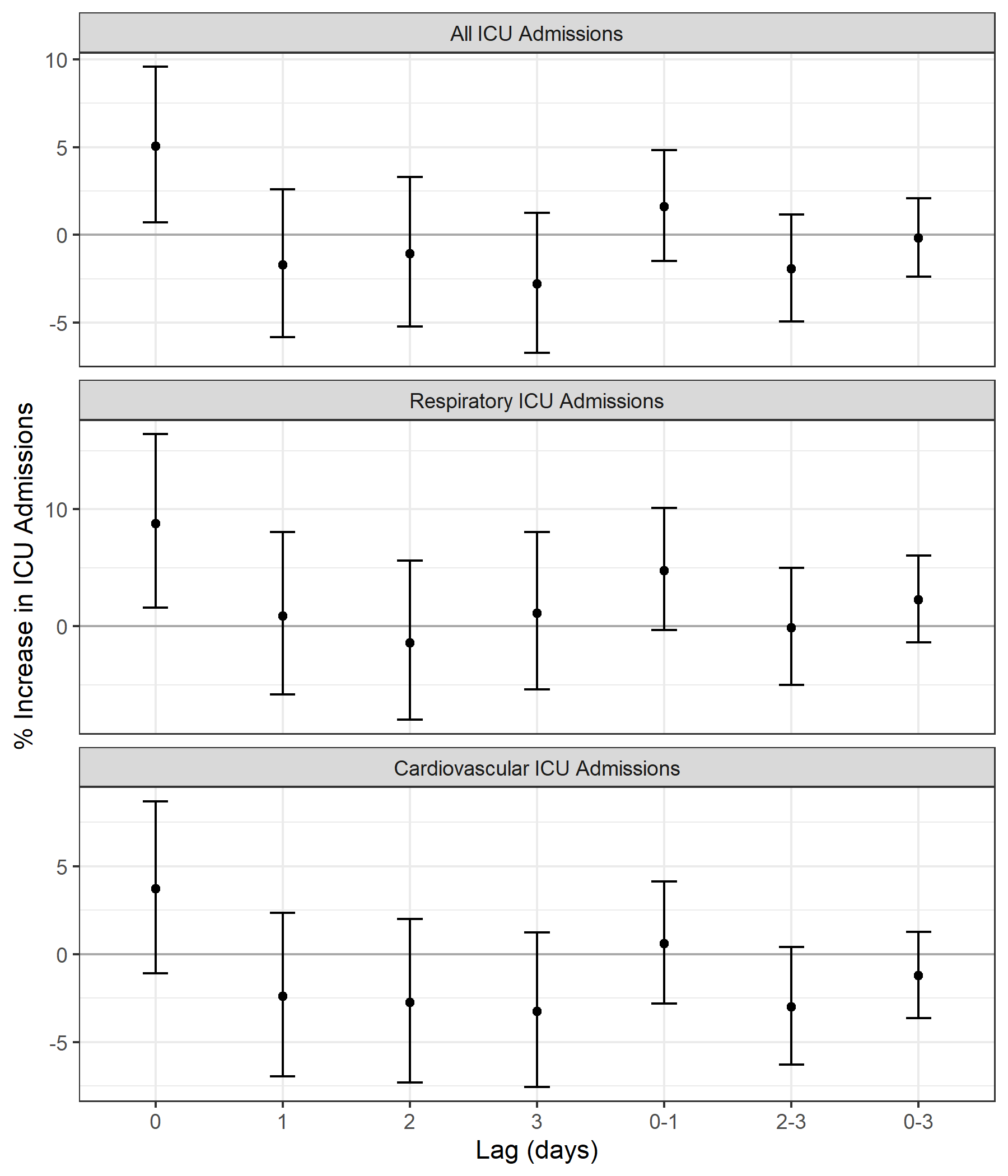


Figure S4: Lagged effects modeled at 0-4 days.


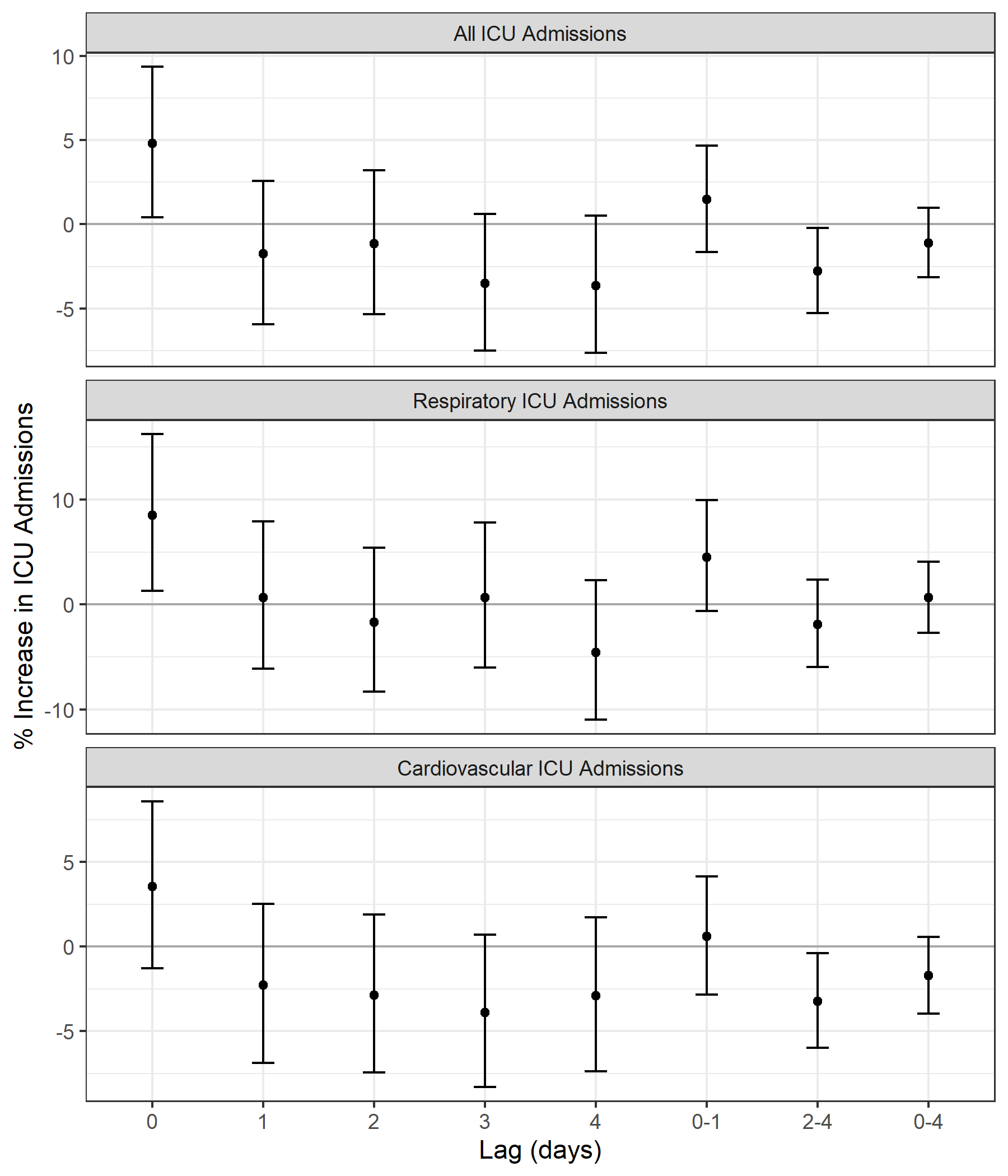


Figure S5: Sensitivity analysis of main results given different buffer distances around meteorological and air pollution monitors.


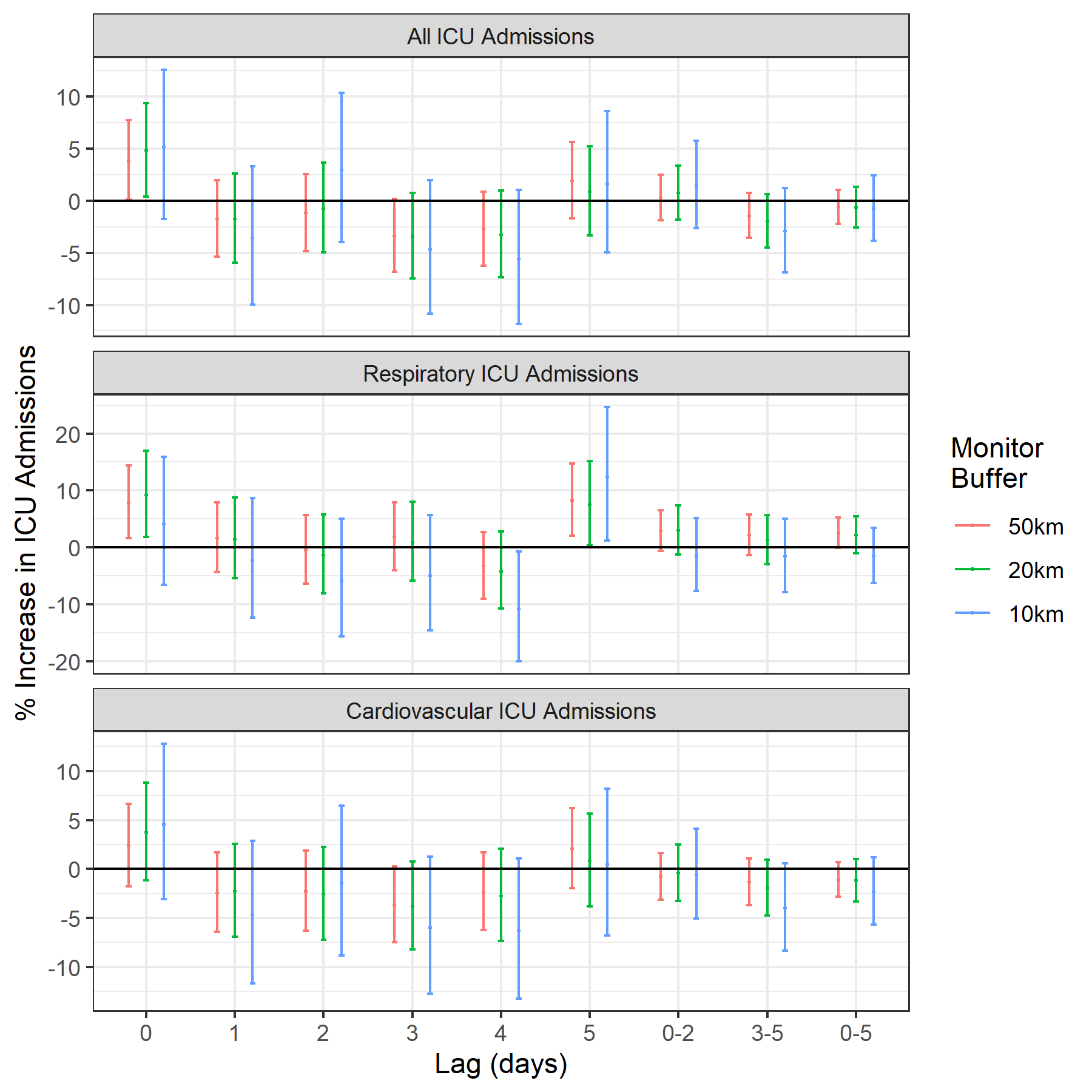


Figure S6: Sensitivity analysis of main results given different buffer distances around Weather Forecast Zones (WFZ).


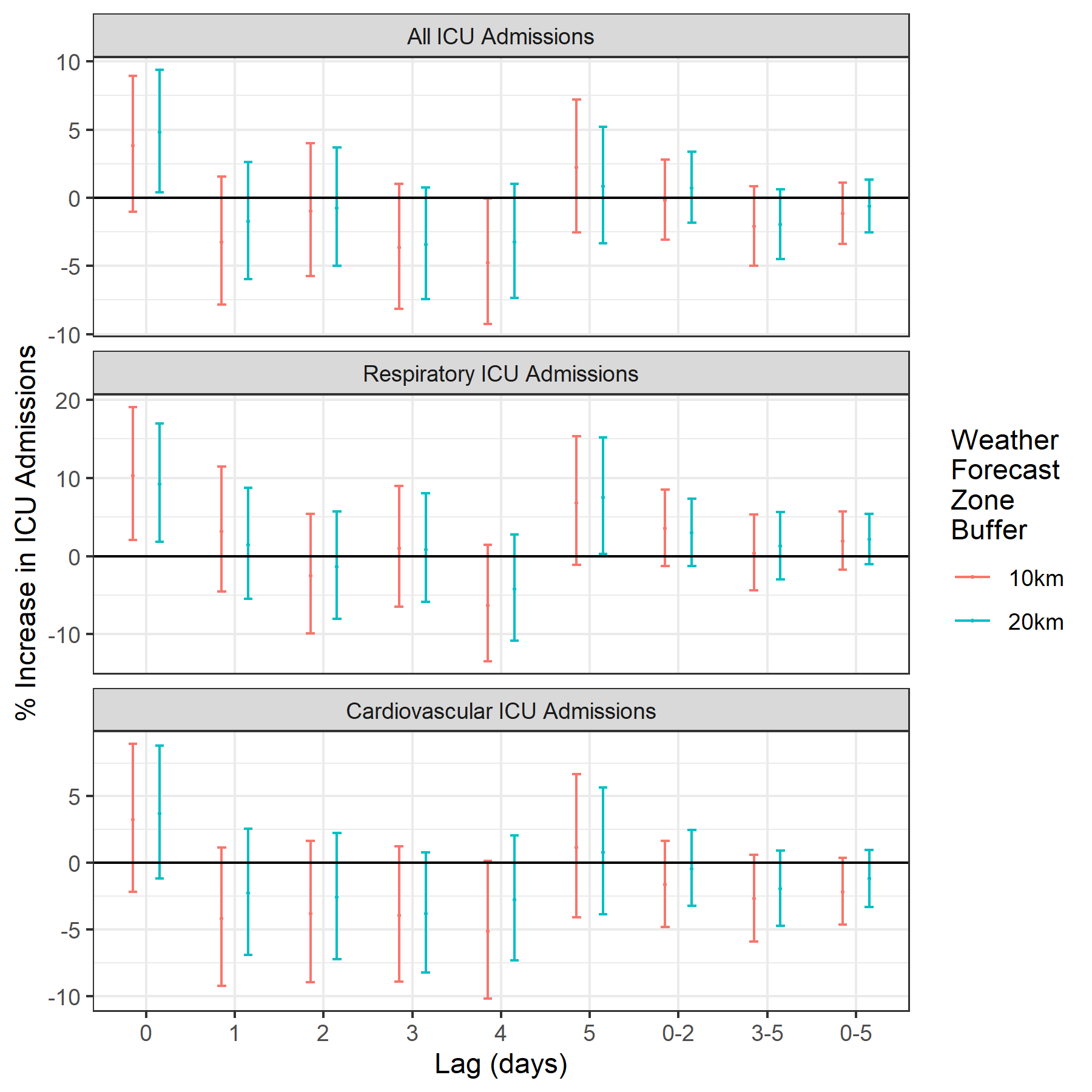

Supplement: Supplementary file 1 — Supporting Information S1 [file GH2-4-e2020GH000260-s001.docx]
